# Patient-reported outcomes and health-related quality of life in individuals living with, through, and beyond cancer in Sweden: a cross-sectional study

**DOI:** 10.1186/s41687-026-01115-z

**Published:** 2026-06-03

**Authors:** Sixten Borg, Linn Rosell, Lars Garpenhag, Susanna Calling, Anna-Maria Larsson

**Affiliations:** 1 Regional Cancer Centre South, Lund, Sweden; 2 Department of Health Sciences, Lund University, Lund, Sweden; 3 Division of Psychiatry, Department of Clinical Sciences in Lund, Lund University, Lund, Sweden; 4 Center for Primary Health Care Research, Department of Clinical Sciences in Malmö, Lund University, Lund, Sweden; 5 Office for Primary Care, Skåne University Hospital, Lund, Sweden; 6 Division of Oncology, Department of Clinical Sciences in Lund, Lund University, Lund, Sweden; 7 Department of Hematology, Oncology and Radiation Physics, Skåne University Hospital, Lund, Sweden

**Keywords:** Patient-reported outcome measures (PROM), EORTC QLQ-C30, Quality-Adjusted Life Years (QALY), Clinical relevance, Breast cancer, Colorectal cancer, Lung cancer, Prostate cancer

## Abstract

**Background:**

The use of patient-reported outcome measures (PROM) can be beneficial in several ways. However, presentations of PROM data from individuals living with, through, or beyond cancer are scarce in the existing scientific literature, and challenges in presenting and interpreting PROM data may be an explanation. Our overall aim was to describe aspects of wellbeing in individuals living with current or previous cancer, 1–2 and 5–6 years after diagnosis. We also aimed to identify vulnerable subgroups of individuals and to facilitate the interpretation of PROM data by combining different presentation forms.

**Methodology:**

Adults in the Southern healthcare region in Sweden with invasive breast, prostate, lung, or colorectal cancer diagnosed 1–2 or 5–6 years ago were invited to a survey using the European Organisation for Research and Treatment of Cancer (EORTC) instrument QLQ-C30 (C30). We analyzed subgroups of respondents using scale scores, clinically anchored problem ranges, and Quality-Adjusted Life Years weights. Moreover, we compared our C30 scores to Swedish general population reference values, EORTC diagnose-specific reference values, and other similar cancer studies.

**Results:**

A total of 2,131 individuals responded (26% response rate). The results demonstrate consistent differences in C30 scale scores, where respondents with several comorbidities, younger age, and lung and colorectal cancer diagnoses had poorer scores. Respondents within problem ranges were consistent with the results on scale scores. Quality-Adjusted Life Years weights displayed a generally similar pattern. Our respondents showed no differences on C30 scales compared to the Swedish general population and recent similar studies. There were some differences compared to diagnose-specific reference values and some older similar studies.

**Conclusions:**

Aspects of wellbeing varied between subgroups, suggesting potential to develop care for certain patient groups with poorer scores, i.e. the younger age groups and in those with several comorbidities, and individuals diagnosed with lung cancer and colorectal cancer. However, individuals who lived 1–2 or 5–6 years after cancer diagnosis experienced no difference in wellbeing than published results from the Swedish general population. Our different ways of presenting PROM show similar results overall but serve to elucidate different aspects which may contribute to the practice of presenting and interpreting PROM data.

**Supplementary Information:**

The online version contains supplementary material available at 10.1186/s41687-026-01115-z.

## Background

Patient-reported outcome measures (PROM) can be used to create benefit in several ways, by giving a voice to the patient and letting healthcare professionals participate further in the patient’s experiences [1, 2]. It can facilitate patient engagement and participation in decisions and care [3, 4]. We can measure outcomes, detect differences, and changes [1, 5]. This, in turn, can detect variations in patient experiences and identify patient groups with vulnerabilities [1, 4, 6]. Finally, we can measure burden of illness, inform decisions, and set priorities [4, 6, 7].

Guidelines recommend the use of PROMs, e.g., to get a comprehensive view of health status, identify health disparities, assess performance of healthcare systems [6, 8], and collect data for cancer quality registers [1]. According to the cancer plan of the Southern Healthcare Region in Sweden, healthcare organizations should regularly take stock of patients´ experiences and needs using PROM [9]. However, how data is presented and used in a clinical setting differs significantly, and PROM data are scarce in the literature from individuals living with, through, and beyond cancer in Sweden.

The Cancer survivors in Primary care Southern Sweden (CAPR1S) project studies individuals living after a cancer diagnosis, and their pathway from specialized cancer care to primary care. Specifically, individuals diagnosed with any of the four common cancer types, breast, colorectal, lung, or prostate cancer, were included 1–2 or 5–6 years after diagnosis. These individuals can thus be said to live with, through, or beyond cancer [10]. The scope of CAPR1S includes various aspects of wellbeing such as functioning, symptoms, health-related quality of life, as well as treatment status, expressed need for healthcare, satisfaction with care, and barriers. We used a questionnaire to cover these aspects; thus, all variables except age, sex, cancer diagnosis, and time since diagnosis are self-reported. Due to the many aspects addressed, respondent burden became a concern. The wellbeing aspects were addressed using a generic cancer PROM instrument with various scales for functioning, symptoms, health, and quality of life, namely the European Organisation for Research and Treatment of Cancer (EORTC) Core Questionnaire QLQ-C30 [11], henceforth denoted C30. This is a frequently used instrument in the field of cancer, e.g., in clinical trials and for follow-up of care in some 10 cancer quality registers in Sweden. We looked for studies using C30 to measure aspects of wellbeing in individuals living with any of above-mentioned four cancer diagnoses in Sweden, one year or more after diagnosis. Although C30 is commonly used, we found only a handful with different aims and scopes with regard to diagnoses, time since diagnosis and participant age span [12–15].

Presenting and interpreting PROM data, including scores from C30, is not entirely straightforward [4], and this may have contributed to the scarcity of data in the literature. Sharing information between different stakeholders enhances the value of PROM data and reduces the reporting burden on the patient, but it requires the ability to interpret the data [16]. One difficulty is that there are few recognizable reference levels for PROM, like there are for physical measurements. There are, however, reference values for C30 from the Swedish general population [17, 18]. EORTC has diagnose-specific reference values from patients pooled from several countries [19]. EORTC supplements these reference values with distributional data to aid comparisons when analyzing and reporting results [19]. A manual for using EORTC measures in clinical daily practice suggests using colours to indicate poor and better levels, and relating scores to normal population percentiles. This manual notes a lack of guidelines, suggesting further development of presentation and interpretation of PROM data, preferably context-dependent for different needs and audiences [20]. Both statistical significance and clinical relevance are important when judging, e.g. estimated differences [21, 22], and there are guidelines for categorizing clinical relevance of cross-sectional differences between groups [23]. Furthermore, for most of C30’s scales, there are clinically anchored problem ranges defined by worries, need for help or care, or limitations in daily life [24]. Finally, C30 can be mapped so that Quality-Adjusted Life Years (QALY) can be estimated [25, 26]. A QALY is a generic measure where time is weighted according to its quality (QALY weight). QALYs enable comparisons across different diagnostic areas and are used by stakeholders and authorities in decision-making and setting priorities [27, 28]. Of specific interest, the use of QALYs enables the combination of many PROM aspects into an overall preference-based measure.

Given the difficulties, but also the availability of several useful resources, there seems to be an opportunity to deepen the knowledge gap in how to present and interpret PROM data. We combined a set of existing methods to contribute to the practice of presenting and interpreting PROM data.

### Aims

Our aim was to describe different aspects of wellbeing in individuals living with current or previous cancer 1–2 and 5–6 years after diagnosis, in terms of the PROM instrument EORTC QLQ-C30 covering functioning, symptoms, and health-related quality of life, and proportions of individuals within problem ranges, and QALY weights. We aimed to identify vulnerable subgroups of individuals. To facilitate interpretation of the PROM data, we used multiple analytical methods and compared our results to available reference values and relevant studies.

## Material and methods

### Material

Using the Swedish National Cancer register, we invited adult (18+) residents in the Southern healthcare region, diagnosed with invasive breast, colorectal, lung or prostate cancer 1–2 or 5–6 years earlier, to participate in an online questionnaire [29].

Cancer register data on age, sex, diagnosis, date of cancer diagnosis, region, address, and vital status were complemented with questionnaire data covering functioning, symptoms, and health-related quality of life. We grouped the respondents by diagnosis, time since diagnosis (1–2, and 5–6 years, respectively), sex, and age (18–49, 50–59, 60–69, 70–79 and 80 + years, respectively).

Data on comorbidities were collected as self-reported presence of high blood pressure, cardiovascular disease, diabetes, chronic obstructive pulmonary disease, asthma, thyroid disease, dyspepsia, irritable bowel disease, pain, anxiety/depression, or other chronic illness, according to previous literature on comorbidities [30]. We categorized each patient based on the number of reported comorbidities, in groups with 0, 1, 2, and ≥ 3 comorbidities, in addition to the cancer diagnosis.

The questionnaire data were collected using the REDCap system [31]. Analyses were carried out using R 4.4.2 [32] and RStudio 2024.12.1 [33].

### The C30 instrument

We used the PROM instrument C30 [11], version 3 [34], to assess functioning, symptoms, and health-related quality of life. C30 contains 30 items, grouped into a number of scales ranging from 0 to 100. Functional scales indicate better functioning with higher values, while symptom scales indicate worse symptoms with higher values (Table 1).

**Table 1:** C30 Functional and symptom scales and their respective scope

Functional scale	Scope
Physical functioning	Carry heavy things, walking, need to sit down or be confined to bed, and need help with dressing, eating, hygiene.
Role functioning	Carry out daily e.g. work or leisure activities.
Emotional functioning	Anxiety, worry, irritability, depression.
Cognitive functioning	Concentration, memory.
Social functioning	Family life, social activities.
General quality of life	Self-reported health and quality of life.

**Symptom scale**	**Scope**
Fatigue	Need to rest, feeling weak or tired
Pain	Pain, and if pain affected daily activities
Dyspnea*	Shortness of breath
Insomnia*	Difficulties sleeping
Appetite loss*	Appetite loss
Nausea/vomiting	Nausea and vomiting
Diarrhea*	Diarrhea
Constipation*	Constipation
Financial problems*	If treatment or condition led to financial difficulties

Notes: * single-item scales. Scale names according to Björdal, de Graeff et al. [34]

### Scale scores and differences

C30 scale scores were computed according to guidelines [21], and presented descriptively overall and by patient subgroups, i.e. by sex, age, cancer diagnosis, time since diagnosis, and number of comorbidities. Generally, interpretation of PROM scores follows two main routes, relating them to either distribution-based or clinically anchored thresholds [21, 22, 24, 35–37]. Differences between groups of patients can be detected using statistical tests or anchor-based methods [38]. Statistical significance does not guarantee clinical relevance [21]. On the other hand, a clinically relevant difference is not necessarily statistically significant [22]. Therefore, it is reasonable to require that a difference is both clinically relevant and statistically significant, as proposed for changes over time [37]. The literature mainly concerns changes over time, but there are also guidelines for categorizing the clinical relevance of differences between groups [23, 36]. Thus, we adopted the approach that a relevant difference in scale scores between groups is required to be both statistically significant and clinically relevant. Mean scores were tested with t-tests between two groups and analysis of variance in the case of more groups. We considered p-values < 0.05 as statistically significant. The clinical relevance of scale score differences between groups were categorized into trivial, small, medium, and large [23]. We used the categorization with slight amendments. For the scales Diarrhea and Financial problems, where medium and large could not be separated due to lack of data [23], we categorized only into trivial, small, and medium. They provided no guidelines for the scale Emotional functioning because estimates did not separate between the non-trivial levels. Therefore, we only categorized differences on this scale as trivial and non-trivial. The thresholds for magnitudes of differences are presented in Supplementary Table S1. Finally, we defined a meaningful difference as a statistically significant difference of size medium or large. Our definition is applicable to all C30 scales except Emotional functioning, on which we refrain from defining meaningful differences.

### Problem ranges

Giesinger et al. identified problem ranges for each C30 scale except General quality of life [24]. The range reflects expected scores for patients meeting at least one of the following criteria: experiencing limitations in daily life, need for help or care, or the patient self or family/partner feeling worried, with regards to the scope of the scale. The ranges are defined by scale score thresholds (Supplementary Table S2). We computed the proportion of respondents within the problem range for each scale and compared these proportions between the different subgroups defined above.

### QALYs and QALY-weights

In a QALY, time is weighed according to its quality, using weights ranging between 0, representing death, and 1, representing perfect health [7]. E.g., a life year spent in perfect health generates one QALY, whereas a life year with a weight of 0.5 generates 0.5 QALYs, and no QALYs are generated after death. QALY weights were estimated to obtain a generic measure of quality of life comparable across disease areas. We used response mapping from C30 to EQ-5D [25], and we then applied a Swedish experience-based value set for EQ-5D [26]. Although the mapping procedure introduces uncertainty in the intermediate step of predicting EQ-5D responses, it produces the QALY weight, which is a single overall preference-based measure derived from many PROM aspects (see Discussion). We tested QALY weight differences between subgroups (as defined above) using t-tests between two groups and analysis of variance between more than two groups.

### Previously published PROM data

We compared our scale scores to previously published reference values for the C30 scales obtained from the Swedish general population, which were collected in 1997 [17] and 2008 [18]. The reference values were weighted according to the demographic structure (sex and age) in our material. We used the first set of reference values as our primary comparator due to its wider age range and higher level of detail [17], and the more recently published values in a sensitivity analysis [18].

We also compared our diagnose groups to diagnose-specific reference values from EORTC [19]. These are official C30 reference values; however, only 4% of the patients were Swedish.

Therefore, as a supplement, we also compared our material to the studies of individuals living with cancer that we found in the literature. Borghede et al. collected PROM in 1994 to study individuals 2–4 years after they were diagnosed with prostate cancer [12]. They used a previous version of C30, excluding the scale Physical functioning from comparison. In 2000–2001, Thomé et al. studied individuals with a prevalent mix of cancers several years after diagnosis [13]. Wikman et al. studied health-related quality of life in the general population in Sweden, covering a limited sample of prevalent long-term cancer in elderly individuals [14]. Sjövall et al. presented C30 data from 2014 to 2018 from individuals in the quality register for colorectal cancer, one year after diagnosis [15]. Necessary data for statistical testing were not presented in Sjövall et al., limiting comparisons to clinically relevant differences. We also used a large registry-based Danish study as a comparator, examining health-related quality of life in Danish cancer survivors of four major cancer types, quite similar to our study [39].

We tested the differences in mean scale scores to the above reference value sets and comparators using normal approximation [40], and flagged any identified meaningful differences using the approach described above.

## Results

### Study population

The overall response rate was 26%, ranging between 35% in individuals aged 50–59 years to 12% in ≥ 80 years old. The age range of the respondents was 26–91 years. The study population, i.e. responders *n* = 2 131, is summarized in Table 2.

**Table 2:** Study population, number of participants by subgroup

	Study participants *n* (%)
**Gender**	
Men	1 196 (56)
Women	935 (44)
**Age (years)**	
18–49	146 (7)
50–59	327 (15)
60–69	644 (30)
70–79	817 (38)
80–	197 (9)
**Diagnosis**	
Breast cancer	720 (34)
Prostate cancer	906 (43)
Lung cancer	100 (5)
Colorectal cancer	405 (19)
**Time since diagnosis**	
1–2 years	1 278 (60)
5–6 years	853 (40)
**Number of comorbidities**	
None	705 (33)
One	726 (34)
Two	391 (18)
Three or more	309 (15)
**Total**	2 131 (100)

### Scale scores and differences between subgroups

We present the scores on functional scales (Table 3) and symptom scales (Table 4) for our study population overall and subdivided into subgroups by sex, age, diagnosis, time since diagnosis, and number of comorbidities. Within each set of subgroups, we examined whether there was a meaningful difference between any pair of subgroups. We found a number of meaningful differences on functional scales (Table 3), as well as on symptom scales (Table 4).

**Table 3:**
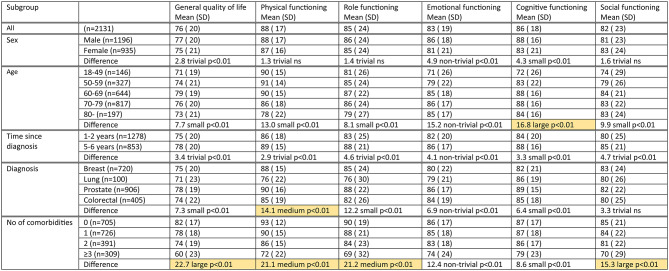
C30 Functional Scale scores (0-100; 100 = best score) overall and by subgroups

Notes: *Emotional function: categories small, medium and large combined, denoted non-trivial. ** *p* < 0.05 and at least medium size difference (See Methods)

P-value and clinical significance indicated for largest detected difference between pair of subgroups*. Meaningful differences indicated with background colour**

**Table 4:**
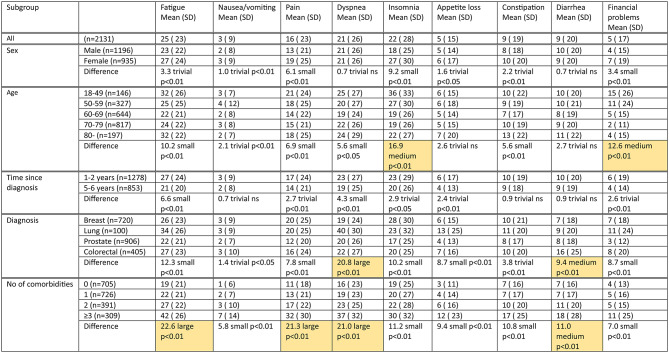
C30 Symptom Scale scores (0-100; 0 = best score) overall and by subgroups

Notes: * Financial problems, Diarrhea: categories medium and large combined, denoted medium. ** *p* < 0.05 and at least medium size difference (See Methods)

P-value and clinical significance indicated for largest detected difference between pair of subgroups*. Meaningful differences indicated with background colour**

**Fig. 1:**
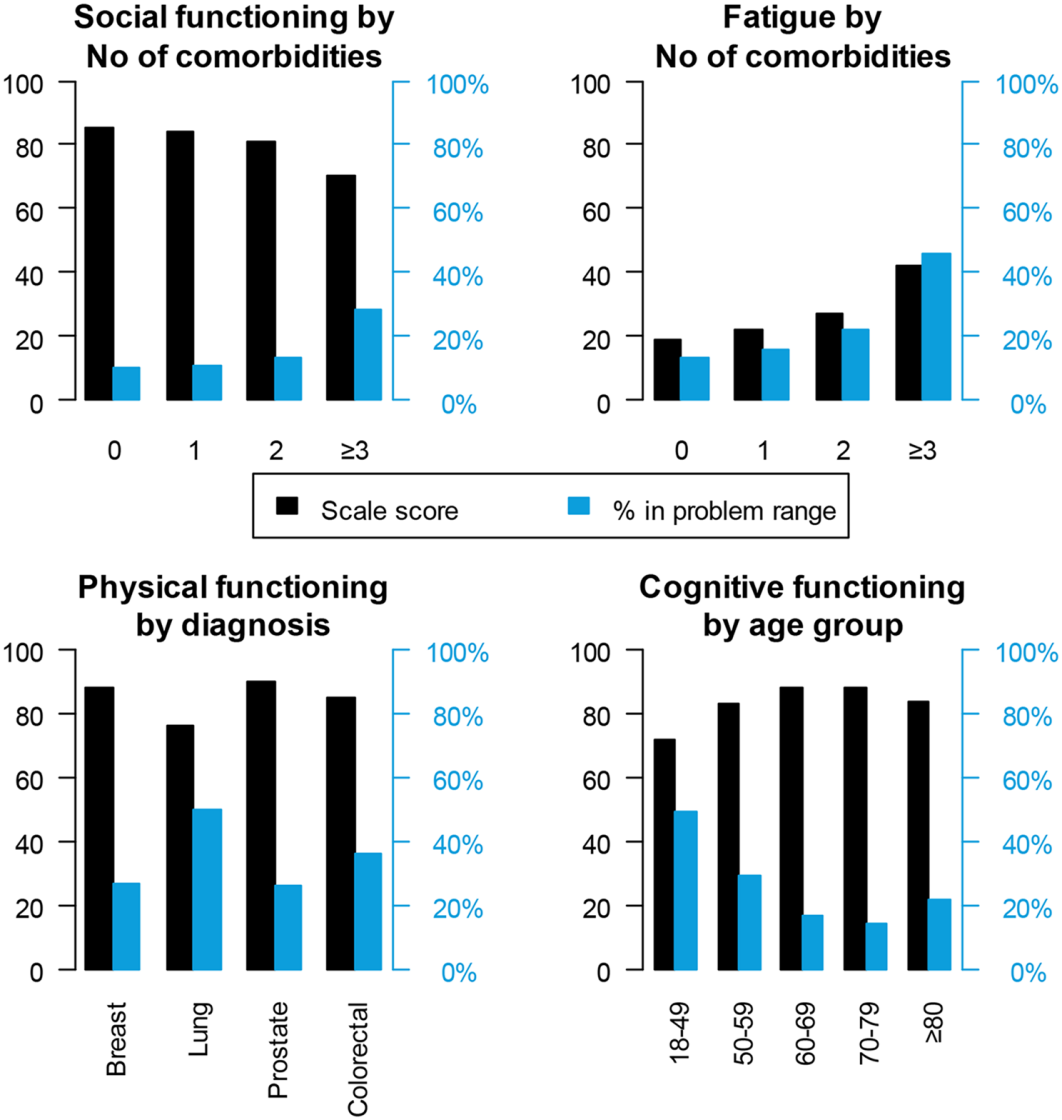
Social functioning and Fatigue by number of comorbidities, Physical functioning by diagnosis and Cognitive functioning by age group. Notes: High scores indicate high functioning, or high level of fatigue, respectively. % is the share of respondents experiencing limitations in daily life, need for help or care, or worry, within the scope of the scale [24]

The subgroup with ≥ 3 comorbidities reported a large difference in General quality of life compared to respondents with 0–1 comorbidities, and medium compared to respondents with two comorbidities. Furthermore, the group with ≥ 3 comorbidities reported a worse score on Physical functioning than the other groups (medium difference). A higher number of comorbidities was associated with poorer scores on Role functioning and Social functioning (Fig. 1), as well as Fatigue, Pain, Dyspnea, and Diarrhea.

Regarding diagnosis, there was a medium difference in Physical functioning between lung and prostate cancers, with prostate scoring better (Fig. 1). Dyspnea was worse in lung cancer, with large differences compared to the other diagnoses. Diarrhea was worse in colorectal cancer, with a medium difference compared to the other diagnoses. There were no meaningful differences associated with time since diagnosis on any of the C30 scales.

Finally, looking at age groups, the worst score in Cognitive functioning was reported in the group 18–49 years, with large differences compared to age groups 60–69 years and 70–79 years; medium difference compared to the oldest group (Fig. 1). Insomnia and Financial problems were worse among the youngest, 18–49 years, compared to 60–69, 70–79, and ≥ 80 years (medium differences).

### Respondents within problem range

When comparing all subgroups, respondents with ≥ 3 comorbidities had the highest proportion within problem range on Physical functioning, Role functioning, Social functioning, Fatigue, Nausea/vomiting, Pain and Constipation (Table 5; Fig. 1).

**Table 5:** Proportion of respondents within problem range (limitations in daily life, need for help or care, or worry, within the scope of the scale), overall and by subgroup

Subgroup	Physical functioning	Role functioning	Emotional functioning	Cognitive functioning	Social functioning	Fatigue	Nausea/ vomiting	Pain	Dyspnea	Insomnia	Appetite loss	Constipation	Diarrhea	Financial problems
All (*n* = 2131)	28%	12%	21%	20%	13%	21%	11%	27%	48%	15%	3%	5%	21%	11%
Male (*n* = 1196)	26%	11%	17%	16%	13%	17%	9%	23%	48%	11%	2%	4%	22%	8%
Female (*n* = 935)	29%	13%	27%	26%	14%	25%	14%	33%	47%	20%	3%	6%	20%	15%
Age 18–49 (*n* = 146)	21%	14%	**42%**	**49%**	23%	33%	12%	36%	55%	**28%**	3%	8%	23%	**29%**
Age 50–59 (*n* = 327)	20%	13%	28%	29%	18%	25%	15%	31%	43%	20%	4%	5%	23%	20%
Age 60–69 (*n* = 644)	23%	9%	19%	17%	11%	16%	9%	24%	45%	12%	2%	3%	19%	11%
Age 70–79 (*n* = 817)	30%	12%	18%	14%	11%	19%	11%	26%	49%	12%	2%	5%	22%	5%
Age 80- (*n* = 197)	50%	19%	20%	22%	13%	27%	11%	30%	51%	15%	6%	9%	23%	8%
1–2 years (*n* = 1278)	30%	14%	24%	23%	15%	24%	12%	29%	51%	16%	3%	6%	22%	13%
5–6 years (*n* = 853)	24%	9%	18%	16%	10%	15%	10%	25%	43%	12%	2%	4%	21%	9%
Breast (*n* = 720)	27%	13%	27%	28%	13%	26%	14%	33%	46%	20%	2%	7%	16%	14%
Lung (*n* = 100)	50%	21%	28%	18%	18%	33%	13%	36%	**78%**	18%	**11%**	6%	21%	22%
Prostate (*n* = 906)	22%	9%	17%	13%	12%	14%	8%	21%	45%	11%	2%	3%	19%	6%
Colorectal (*n* = 405)	36%	15%	21%	23%	16%	23%	12%	29%	49%	11%	3%	6%	**36%**	16%
No comorbidities (*n* = 705)	16%	7%	16%	18%	10%	13%	7%	19%	37%	11%	1%	3%	16%	8%
One comorbidity (*n* = 726)	21%	9%	19%	17%	11%	16%	8%	22%	46%	13%	2%	3%	17%	10%
2 comorbidities (*n* = 391)	35%	13%	22%	21%	13%	22%	12%	32%	53%	16%	3%	6%	27%	12%
≥ 3 comorbidities (*n* = 309)	**62%**	**31%**	39%	34%	**28%**	**46%**	**28%**	**53%**	69%	23%	7%	**13%**	36%	20%

Note: highest proportion (before rounding) indicated with boldface

The youngest age group had the highest proportion within the problem range on Emotional functioning, Cognitive functioning, Insomnia, and Financial problems.

Respondents with lung cancer had the highest proportion within the problem area on Dyspnea and Appetite loss, whereas colorectal cancer had the highest proportion on Diarrhea.

Across all scales, Appetite loss had the lowest proportion within problem range, 3%, and Dyspnea had the highest, 48%, nearly half the study population. The proportion was 78% in the lung cancer subgroup. Among respondents with the comorbidities chronic obstructive pulmonary disease and asthma, 85% and 68%, respectively, were within the problem range of Dyspnea.

### QALYs

The mean QALY weight in our study population was 0.87. It differed by age group, with the lowest weight in ≥ 80 years (0.84) and highest in 50–69 years (0.88), please see the discussion section, and by time since diagnosis (0.86 in 1–2 years, 0.88 in 5–6 years). It also differed by diagnosis, where respondents diagnosed with lung cancer had the lowest weight (0.83) compared to prostate cancer (0.88), breast cancer (0.87) and colorectal cancer (0.86). The weight also differed by number of comorbidities, higher in respondents without any (0.89) compared to respondents with 1, 2, and ≥ 3 comorbidities, respectively (0.88, 0.86, 0.80).

### Comparison to other sources

We found no meaningful differences between our study population and the Swedish general population reference values on any scale (Supplementary Table S3). Nor did we find any differences compared to the reference values in subgroups of our study population by time since diagnosis (data not shown). The results were the same in all these regards using the alternative set of general population values.

Our respondents with either breast, lung, and colorectal cancer reported better scores on General quality of life than the corresponding diagnose-specific EORTC reference populations (Supplementary Table S3). In addition, respondents with breast cancer reported a better score on Financial problems, and respondents with lung cancer reported a better score on Appetite loss. These all comprise meaningful differences.

Compared with other studies of individuals living with, through, or beyond cancer, our prostate cancer group reported a meaningfully better score on Social functioning, though comparator data were from 1994 (Supplementary Table S4). Overall, our population reported what we defined as meaningfully better scores on General quality of life, Role functioning, Physical functioning, Fatigue, Pain, and Dyspnea compared to a comparator from 2000 to 2001, but no meaningful differences were detected compared to a comparator from 2008. Finally, no differences were detected between our colorectal cancer group and comparator data from 2014 to 2018.

## Discussion

In this study, we present PROM data collected using C30 to describe different aspects of wellbeing in individuals diagnosed with invasive breast, colorectal, lung, or prostate cancer, 1–2 or 5–6 years ago. In an effort to facilitate the interpretation of our PROM data, we estimated and presented functional and symptom scale scores, computed the proportions of respondents within problem ranges, derived QALY weights, and finally compared scale scores to reference values and other studies. We used subgroup analyses based on age, sex, diagnosis, time since diagnosis and number of comorbidities to identify vulnerable subgroups among our respondents.

When examining subgroups some general patterns appeared across our different data presentations. Respondents with several comorbidities reported poorer functioning and worse symptoms, higher proportions within problem ranges, and lower QALY weight. We saw poorer wellbeing associated with specific diagnoses, e.g. Physical functioning and Dyspnea with lung cancer, and Diarrhea with colorectal cancer, both in terms of scale scores and proportions within problem range. Respondents with lung cancer also had the lowest QALY weight. The youngest age group reported the worst wellbeing in terms of scale scores and proportions of respondents within the problem range, but the oldest age group had the lowest QALY-weight. Age is a factor in the response mapping we used, if only with a modest impact, but the results involving age should be interpreted with care. Regarding time since diagnosis, the subgroup 5–6 years had a higher QALY-weight than the 1–2 years group. However, no associations with time since diagnosis were detected on the individual C30 scales. This difference detected in the QALY weight may stem from aggregating small undetected differences on individual C30 scales. The QALY weight difference suggests that these different aspects tend to improve as time passes since diagnosis.

Using two sets of data from the general Swedish population [17, 18], adjusted to match the sex and age structure of our study population, we found no meaningful differences on any C30 scale compared to any of them. Michelson et al. examined a subgroup of individuals with a prevalent mix of cancer diagnoses, and found statistically significant differences between this cancer group and their general population values [17]. Wikman et al., sharing general population values with Derogar et al. [14, 18], also found differences between their group with prevalent mix of cancer and their general population values. We had a much larger sample and thus higher statistical power and indeed found statistically significant differences between our study participants and each of these general population value sets (Supplementary Table S3), but we also chose a more conservative approach focusing on meaningful differences, namely both statistically significant and clinically relevant.

Comparing our study population to EORTC’s diagnose-specific reference sets (Supplementary Table S4), we saw that the participants with breast, lung, and colorectal cancer reported better functioning than the corresponding reference sets (General quality of life), and these were meaningful differences. Our data were collected in 2023, whereas the reference data were collected from 1993 to 2007, and we would expect many changes in cancer diagnostics, treatment, and care during the time in between. In addition, there are relatively few Swedish patients in these reference data, which could impact the results due to nation-specific differences in cancer care and wellbeing.

Comparing our study population to other studies of individuals with cancer revealed meaningful differences compared to those from 1994 (Social functioning) [12], and 2000–2001 (e.g. Physical functioning) [13], but not compared to those from 2008 and 2014–2018 [14, 15] (Supplementary Table S3). Levinsen et al. carried out a study similar to ours, where breast, lung, prostate, and colon cancer survivors identified in the Danish cancer registry were invited to participate, although their focus was HRQOL by educational level [39]. Using the same problem ranges as we did [24], they saw more symptoms and poorer functioning in lung and breast cancer than in colon and prostate cancer. We did not see this in general, but we saw that breast and lung cancer participants had the highest proportion within the problem range on the scales Emotional functioning, Fatigue, and Pain. Levinsen et al. had a significantly larger sample, some 28,000 patients, hence larger power than we had. Unlike in our study, they excluded patients in active cancer treatment and collected data on a population where the majority had been diagnosed 5–12 years earlier. These differences may have contributed to our slightly different results.

To summarize, the sources for these comparisons come from a wide time span from 1993 to 2023 and represent different types of cancer, origins of patients, and settings, e.g. looking back longer in time, more differences were detected. Given that the general population data were collected in 1997 and 2008, respectively, and findings that self-rated health and anxiety change over time in the general population [41, 42], a collection of a new set of general population reference values may be needed to properly compare with current data. Furthermore, several changes related to Swedish cancer care occurred within this time span. Cancer survival has improved in Sweden, including the four cancer types presented here [43]. This could be the result of national clinical care guidelines with uniform therapeutic recommendations, implementation of standardized cancer care pathways, introduction of new drugs, therapies, and initiatives facilitating early discovery [43]. This may have affected the severity of symptoms and health consequences during and after cancer, or while living with a suppressed illness. This, in turn, may have affected our comparisons to the general population and other sources.

Turning focus to analytic methods, we presented PROM data in different ways to aid the interpretation of our PROM data. We benefitted from previous work on identifying clinically relevant differences [23, 37] and clinically relevant problem ranges [24], and various reference values [17–19]. Furthermore, what constitutes an important difference (e.g. minimal important difference) varies between scales, contexts, and other circumstances, and there are recommendations to rely not on a single source but use all available guidelines at hand [22]. We tried to see which patterns appear when we used clinically relevant thresholds [23] combined with statistical significance [37], and clinically anchored problem ranges [24], as well as overall preference-based measurements in the form of QALY-weights [25, 26]. The different aspects of wellbeing we use have their respective scopes, but some are correlated. This does not mean we have a single underlying construct, but that different aspects are still related, for instance pain and insomnia. Although different in scope, they could reasonably affect each other. The QALY weight is an aggregate of many aspects into an overall preference-based measure. It provides a summarizing overall measure without implying a single underlying construct. Our idea was to examine if looking at both individual aspects and an overall measure is helpful in interpreting PROM data. We hope this array of approaches can inspire further work on PROM data presentation and interpretation.

Some of our findings are relevant to policy. We presented descriptive data on QALY-weights, where many PROM scales were aggregated using a preference-based value system into an overall measure. We saw QALY-weight differences ranging from 0.02 over time since diagnosis, to 0.09 across the range of number of comorbidities. Both these differences can be characterized as important. The smaller of these differences could be compared to the difference between not having any problems with self-care, and having problems or being entirely unable [26]. A QALY-weight difference of 0.09, meanwhile, corresponds to the difference between having extreme pain or discomfort and having no pain or discomfort [26].

According to our results, the reported wellbeing is fair, but attention could be given to vulnerable subgroups based on specific aspects of wellbeing, e.g. young respondents, respondents with several comorbidities, and lung and colorectal cancer. This could inform the development of the long-term care of individuals living with, through, or beyond cancer. Thus, our results demonstrate that PROM is an advantageous tool for identifying vulnerable subgroups of patients, and, as previous studies have shown, PROM can support patients’ involvement in decision making and engagement in their care [3, 4]. On an organizational level, PROM could facilitate prioritizing healthcare resources and, by identifying patients’ needs, also enhance the coordination and communication between healthcare providers. The benefits of PROM are well documented, and the use of PROM is recommended in national guidelines, yet the use of PROM in clinical settings differs significantly. This indicates a need for structured follow-up on both a management level and at each healthcare unit to increase and harmonize the use of PROM.

Finally, we see a number of strengths and limitations in our study. Our definition of a meaningful difference, requiring the difference to be both statistically significant and clinically relevant, is conservative. Requiring both may reduce the risk of overinterpreting clinically irrelevant differences or mistaking noise for clinically relevant differences. It may also prevent us from noticing differences worth attention, though not formally detected. This should be kept in mind when comparing our results to other studies. We do, however, present both statistical significance and grading of clinical relevance, so the interested reader can choose a less conservative standpoint. We chose C30 to capture functioning and symptoms in individuals living with, through, or beyond cancer. This instrument has been used extensively, but it was not developed specifically for survivorship-related burden. The newly developed and considerably larger instrument QLQ-SURV100 was developed for disease-free cancer survivors [44]. We chose C30 for its available reference values and other studies allowing comparisons, and because of concerns for respondent burden. However, the instrument choice may be a limitation. Our analysis of QALY weights uses a mapping from C30 to EQ-5D [25] and application of a Swedish experience-based value set [26]. The mapping was based on patients with multiple myeloma, breast cancer, and lung cancer, including two of the four cancer diagnoses in the current study. The authors evaluated their mapping using Dolan’s hypothetical UK tariff [45], and hypothesized that it could be sensitive to the choice of tariff or value set [25]. Our choice of an experience-based Swedish value set could therefore increase uncertainty but should be highly relevant in the setting of our study. The mapping itself via the intermediate instrument EQ-5D adds uncertainty. But it also provides an overall preference-based measure, namely the QALY-weight, which we consider very useful. Our response rate of 26% was relatively low, and the subgroup with the lowest response rate (12%) was the oldest patients aged 80+. This subgroup had a large proportion of individuals within the problem range on Physical functioning and had the lowest QALY weight. We might have a selection problem through non-response tendencies, though hardly among individuals with poor wellbeing given these findings. We did, however, observe a range of different levels of wellbeing despite this potential selection problem, but the limitation should be kept in mind. Slightly related, we adjusted for demographic structure in our comparisons to other populations to compensate for differences in structure. We selected a number of factors to define subgroups, as a way to see which factors are associated with differences in various aspects of wellbeing. These findings inherently depend on our choice of factors and subgrouping, however we believe our choice is reasonable. One such factor was the number of comorbidities, specifically they were self-reported, although not clinically verified. Another limitation is that the aggregation into total number of comorbidities assigns equal weights to all comorbidities, whereas some may have a more severe impact than others. Last, with our cross-sectional design, we can only detect associations, thus no causal findings can be made.

## Conclusions

By using a wide approach to reporting PROM from a study population living with, through, or beyond cancer, we can draw the following conclusions:

- Various aspects of wellbeing were poorer in the youngest individuals and in those with several comorbidities, as well as those diagnosed with lung cancer and colorectal cancer. We suggest caregivers pay special attention to these subgroups.
- According to the studied aspects of wellbeing, respondents who lived 1–2 or 5–6 years after cancer diagnosis did not differ from individuals living with cancer in recent studies, nor from previously published results from the Swedish general population.
- Our different ways of presenting PROM generally show similar results overall, but also serve to elucidate specific aspects (e.g. diagnose specific symptoms) and this may contribute to the practice of presenting and interpreting PROM in the context of cancer care.

## Supplementary Information

Below is the link to the electronic supplementary material.

Supplementary Material 1

## Data Availability

The data that support the findings of this study are restricted by the Swedish Ethical Review Authority in order to protect participants’ privacy.

## Abbreviations

C30: The European Organisation for Research and Treatment of Cancer Quality of Life Group Core Questionnaire
EORTC: The European Organisation for Research and Treatment of Cancer
EQ-5D: The EuroqoL 5 Dimension questionnaire
PROM: Patient Reported Outcomes Measures
QALY: Quality-Adjusted Life Years
