# Supplementary material for: Patient-reported outcomes and health-related quality of life in individuals living with, through, and beyond cancer in Sweden: a cross-sectional study
Source: J Patient Rep Outcomes. 2026 Jun 3;10:95. doi: 10.1186/s41687-026-01115-z (PMC13241336; doi:10.1186/s41687-026-01115-z)
Supplement: Supplementary file 1 — Supplementary Material 1 [file 41687_2026_1115_MOESM1_ESM.docx]

# Material suggested to be in an online supplement

**Table S1**: Thresholds for categorizing cross-sectional differences in mean scale scores between groups according to Cocks, King et al. (23), supplemented by assumption regarding Emotional functioning EF (See Methods). A difference belongs in a category if it is below the indicated threshold (e.g. a NV difference of 15 or more is categorized as large).

| **scale** | **trivial** | **small** | **medium** |
| --- | --- | --- | --- |
| Diarrhea | 3 | 7 | NA |
| Nausea/vomiting | 3 | 8 | 15 |
| Cognitive functioning | 3 | 9 | 14 |
| Dyspnea | 4 | 9 | 15 |
| Financial problems | 3 | 10 | NA |
| General Quality of Life | 4 | 10 | 15 |
| Social functioning | 5 | 11 | 15 |
| Insomnia | 4 | 13 | 24 |
| Fatigue | 5 | 13 | 19 |
| Constipation | 5 | 13 | 19 |
| Pain | 6 | 13 | 19 |
| Physical functioning | 5 | 14 | 22 |
| Appetite loss | 5 | 14 | 23 |
| Role functioning | 6 | 19 | 29 |
| Emotional functioning | 3 | NA | NA |

Notes: NA indicates that a category has been combined with the next higher category; For the scales FI and DI, medium and large cannot be separated, any difference above small is denoted medium. For the scale EF, small, medium and large differences cannot be separated and are denoted non-trivial.

**Table S2**: Thresholds for the problem range on C30 scales. A patient falling below the threshold (<x) on a functional scale, or exceeding the threshold (>x) on a symptom scale is in the problem range on that scale (24).

| **Scale** | **Threshold** |
| --- | --- |
| Physical functioning | <83 |
| Role functioning | <58 |
| Emotional functioning | <71 |
| Cognitive functioning | <75 |
| Social functioning | <58 |
| Fatigue | >39 |
| Nausea/vomiting | >8 |
| Pain | >25 |
| Dyspnea | >17 |
| Insomnia | >50 |
| Appetite loss | >50 |
| Constipation | >50 |
| Diarrhea | >17 |
| Financial problems | >17 |

**Table S3**: Comparison of present study (data collected 2023) to other studies of individuals living with cancer in Sweden*, using EORTC QLQ-C30 functional scales and symptom scales. Differences indicated as better/worse compared to the present study.

| **Year of data collection** | **1994** | **2000-2001** | **2008** | **2014-2018** |
| --- | --- | --- | --- | --- |
|  | Borghede et al. (12)  Prostate cancer**^†^** | Thomé et al. (13)  Mix of cancers | Wikman et al. (14)  Mix of cancers | Sjövall et al. (15)  Colorectal cancer**^†^** |
| Meaningful differences^§^ | Worse: Social functioning. | Worse: General quality of life, Role functioning, Physical functioning, Fatigue, Pain, Dyspnea. | None | None** |
| Statistical differences | Better: Diarrhea.  Worse: General quality of life, Role functioning, Emotional functioning, Cognitive functioning, Social functioning, Fatigue, Nausea/vomiting, Pain, Dyspnea, Insomnia, Appetite loss, Constipation, Financial problems. | Worse: General quality of life, Role functioning, Physical functioning, Cognitive functioning, Fatigue, Pain, Dyspnea, Insomnia, Appetite loss, Constipation, Financial problems. | Better: Social functioning.  Worse: General quality of life, Physical functioning, Role functioning, Emotional functioning, Fatigue, Pain. | *** |

*Criteria for inclusion described in Background. ^§^Statistically significant *and* clinically relevant difference. **^†^**Prostate and colorectal compared to the respective subgroups of the present study. **No clinically relevant differences; hence no meaningful differences. *** cannot be established (required data not presented in publication).

**Table S4**: Summary of comparison of C30 scale scores, study population vs. General population (GP), and EORTC Breast cancer (BC), Lung cancer (LC), Prostate cancer (PC) and Colorectal cancer (CRC) reference values. Meaningful differences indicated as medium or large and coloured background (otherwise trivial, small).

(a) Functional Scale scores (100 = best, 0 = worst)

| Comparison | General quality of life | Physical functioning | Role functioning | Emotional functioning | Cognitive functioning | Social functioning |
| --- | --- | --- | --- | --- | --- | --- |
| Study population vs. | 76 | 88 | 85 | 84 | 86 | 82 |
| GP 1997 | 76 trivial | 84 trivial* | 84 trivial | 84 trivial | 87 trivial* | 90 small* |
| GP 2008 | 76 trivial | 87 trivial* | 88 trivial* | 86 trivial* | 88 trivial* | 91 small* |
| Study BC patients vs. | 75 | 88 | 85 | 80 | 82 | 83 |
| EORTC BC | 62 medium* | 78 small* | 71 small* | 69 non-trivial* | 82 trivial | 77 small* |
| Study LC patients vs. | 71 | 76 | 76 | 79 | 86 | 80 |
| EORTC LC | 57 medium* | 72 trivial | 62 small* | 69 non-trivial* | 82 small* | 71 small* |
| Study PC patients vs. | 78 | 90 | 88 | 86 | 89 | 82 |
| EORTC PC | 68 small* | 80 small* | 83 trivial* | 77 non-trivial* | 83 small* | 80 trivial* |
| Study CRC patients vs. | 74 | 85 | 82 | 84 | 85 | 80 |
| EORTC CRC | 61 medium* | 79 small* | 70 small* | 69 non-trivial* | 85 trivial | 76 trivial* |

(b) Symptom scale scores (0=best, 100=worst)

| Comparison | Fatigue | Nausea/vomiting | Pain | Dyspnea | Insomnia | Appetite loss | Constipation | Diarrhea | Financial problems |
| --- | --- | --- | --- | --- | --- | --- | --- | --- | --- |
| Study population vs. | 25 | 3 | 16 | 21 | 22 | 5 | 9 | 9 | 5 |
| GP 1997 | 22 trivial* | 3 trivial | 21 trivial* | 19 trivial* | 18 small* | 4 trivial* | 6 trivial* | 5 small* | 7 trivial* |
| GP 2008 | 20 trivial* | 3 trivial | 20 trivial* | 17 small* | 18 trivial* | 3 trivial* | 6 trivial* | 6 small* | 4 trivial* |
| Study BC patients vs. | 27 | 3 | 20 | 20 | 28 | 6 | 10 | 7 | 7 |
| EORTC BC | 33 small* | 8 small* | 29 small* | 18 trivial | 30 trivial | 19 small* | 17 small* | 6 trivial | 18 medium* |
| Study LC patients vs. | 34 | 3 | 20 | 40 | 23 | 13 | 11 | 9 | 11 |
| EORTC LC | 41 small* | 11 small* | 30 small* | 38 trivial | 32 small* | 28 medium* | 19 small* | 7 trivial | 17 small* |
| Study PC patients vs. | 22 | 2 | 12 | 20 | 17 | 4 | 8 | 8 | 3 |
| EORTC PC | 27 small* | 5 small* | 23 small* | 17 trivial* | 25 small* | 10 small* | 15 small* | 8 trivial | 9 small* |
| Study CRC patients vs. | 27 | 3 | 16 | 22 | 20 | 7 | 10 | 16 | 8 |
| EORTC CRC | 35 small* | 7 small* | 24 small* | 17 small* | 31 small* | 19 small* | 16 small* | 17 trivial | 14 small* |

Notes: * p<0.01. GP = Swedish General Population. EORTC = European Organization for the Research and Treatment of Cancer (reference values).
